# Spindle Assembly Checkpoint Regulates Mitotic Cell Cycle Progression during Preimplantation Embryo Development

**DOI:** 10.1371/journal.pone.0021557

**Published:** 2011-06-24

**Authors:** Yanchang Wei, Saima Multi, Cai-Rong Yang, Junyu Ma, Qing-Hua Zhang, Zhen-Bo Wang, Mo Li, Liang Wei, Zhao-Jia Ge, Chun-Hui Zhang, Ying-Chun Ouyang, Yi Hou, Heide Schatten, Qing-Yuan Sun

**Affiliations:** 1 State Key Laboratory of Reproductive Biology, Institute of Zoology, Chinese Academy of Sciences, Beijing, China; 2 College of Life Science, Northeast Agricultural University of China, Harbin, China; 3 Department of Veterinary Pathobiology, University of Missouri, Columbia, Missouri, United States of America; Institute of Zoology, Chinese Academy of Sciences, China

## Abstract

Errors in chromosome segregation or distribution may result in aneuploid embryo formation, which causes implantation failure, spontaneous abortion, genetic diseases, or embryo death. Embryonic aneuploidy occurs when chromosome aberrations are present in gametes or early embryos. To date, it is still unclear whether the spindle assembly checkpoint (SAC) is required for the regulation of mitotic cell cycle progression to ensure mitotic fidelity during preimplantation development. In this study, using overexpression and RNA interference (RNAi) approaches, we analyzed the role of SAC components (Bub3, BubR1 and Mad2) in mouse preimplantation embryos. Our data showed that overexpressed SAC components inhibited metaphase-anaphase transition by preventing sister chromatid segregation. Deletion of SAC components by RNAi accelerated the metaphase-anaphase transition during the first cleavage and caused micronuclei formation, chromosome misalignment and aneuploidy, which caused decreased implantation and delayed development. Furthermore, in the presence of the spindle-depolymerizing drug nocodazole, SAC depleted embryos failed to arrest at metaphase. Our results suggest that SAC is essential for the regulation of mitotic cell cycle progression in cleavage stage mouse embryos.

## Introduction

To assure correct segregation of genetic materials into daughter cells, eukaryotic cells employ the SAC mechanism to prevent premature metaphase-anaphase transition until all chromosomes successfully attach to the bipolar spindle with proper tension [1]. SAC consists of ‘sensor’ proteins such as Mad1, Bub1 and Mps1; a ‘signal transducer’, consisting of the mitotic checkpoint complex (MCC), composed of Mad2, Bub3, BubR1 and Cdc20; and an ‘effector’ known as the anaphase promoting complex/cyclosome (APC/C) [2]. Prior to metaphase-anaphase transition, SAC inhibits the ability of Cdc20 to activate the APC/C which stabilizes securin and cyclin B, thus the metaphase-anaphase transition is delayed until all chromosomes establish the correct attachment to the spindle [3]. Once the correct attachment has been established, SAC is inactivated and APC/C-Cdc20 ubiquitinates securin and cyclin B, resulting in the activation of separase. Separase removes the cohesion complex holding sister chromatids together so that the cells can enter anaphase [2], [4], [5].

The SAC is not required in budding yeast, perhaps because these cells enter mitotic progression with correct attachment of kinetochores to microtubules [6], [7], [8]. However, in vertebrate cells SAC is essential for normal mitotic progression [9], [10], [11], [12]. Mice with homozygous null mutations in the SAC (Bub3, BubR1 or Mad2) die at a very early stage of embryogenesis [13], [14], [15], [16]. Thus our understanding of SAC in eukaryotic cells has largely been restricted to the analysis of mice with heterozygous mutations which harbor one null and one wild-type allele. Heterozygous mice can develop normally but are predisposed to spontaneous tumor development. Mice with an expression level of approximate 11% BubR1 are not predisposed to tumors but exhibit premature aging phenotypes, and fibroblasts isolated from these mice showed SAC defects and aneuploidy [17]. Heterozygotes with Bub3 mutants also age prematurely [18]. Furthermore, mouse embryo fibroblasts heterozygous for Bub3, BubR1 and Mad2 all show SAC defects and high levels of aneuploidy [15], [19], [20], [21], [22]. Indeed, in HCT166 cells, reduction of Mad2 protein levels to 70% results in complete abrogation of SAC [23].

The initial suggestion that SAC might not exist in vertebrate oocytes which would explain the high incidence of aneuploidy comes from studies of XO mice, which have only one X chromosome but are fertile and phenotypically female [24]. However, this study has been challenged by the finding that microtubule inhibitors such as nocodazole can block polar body extrusion and the onset of securin proteolysis [25], [26], [27], [28]. Furthermore, injection of Mad2, Bub3 or BubR1 morpholinos, or expression of dominant negative Mad2, Bub1 or BubR1 by microinjection of mRNA encoding the mutant protein lacking the kinase domain leads to an acceleration of meiosis, with high levels of chromosome missegregation and aneuploidy [28], [29], [30]. These results demonstrate that SAC does exist and detects attachment errors to microtubules in mouse oocytes.

Mistakes in chromosome segregation or distribution may result in aneuploid embryo formation, which causes spontaneous abortion, genetic diseases, or embryo death [31]. Embryonic aneuploidies are produced when abnormal chromosomes or their abnormal segregation are present in gametes or early stage embryos [31]. To date, there is no direct evidence showing that SAC is required for the regulation of mitotic cell cycle progression during preimplantation development. Conventional genetic approaches have not been informative regarding SAC function in cleavage stage embryos, because it would be difficult to distinguish between errors that had occurred during meiosis and those that occurred during cleavage stage development [13], [14]. To overcome this problem, we have used RNAi-based gene silencing to deplete Bub3, BubR1, or Mad2 in cleavage stage embryos, enabling us to determine the effect of SAC depletion specifically during cleavage stage development. We also employed overexpression of SAC components from an exogenous Myc_6_-SAC mRNA to study their roles in early cleavage. Our results show that SAC is essential for correct chromosome segregation during preimplantation development.

## Results

### Expression and subcellular localization of Bub3, BubR1 and Mad2 in one-cell embryos

**Figure 1:**
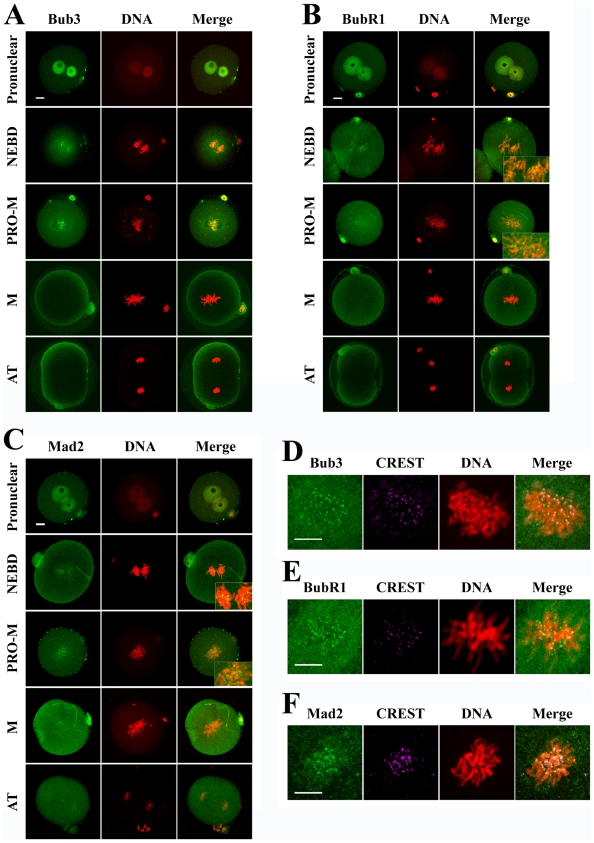
Expression and subcellular localization of SAC components (Bub3, BubR1 and Mad2) in various one-cell embryo stages. (A) One-cell embryos were stained with anti-Bub3 antibody (green) and PI (red). Clear signal of Bub3 was observed at kinetochores in NEBD and pro-metaphase stages. (B and C) Similar as in A, clear signal of BubR1 or Mad2 was also observed at kinetochores in NEBD and pro-metaphase stages. (D, E and F) Co-localization of Bub3, BubR1, Mad2 and CREST at kinetochores. M refers to metaphase, PRO-M refers to prometaphase, and AT refers to anaphase or telophase. Scale bars represent 10 µm.

To investigate the role of Bub3, BubR1 and Mad2, three major components of the MCC, during the first division of preimplantation embryos, we first examined the expression and subcellular localization of these proteins in one-cell embryos by immunostaining. We found that Bub3 protein was mainly localized in the pronuclei at the pronuclear stage; Bub3 signal became detectable at the kinetochores at the time of nuclear envelope breakdown (NEBD); when embryos progressed to pro-metaphase, clear staining was observed at the kinetochores; at metaphase, once all chromosomes were aligned at the equatorial plane, Bub3 signal disappeared from the kinetochores; at the anaphase or telophase stage, the signal for Bub3 was undetectable at kinetochores (Fig. 1A). The signal patterns for BubR1 or Mad2 were very similar to that of Bub3. At the pronuclear stage, BubR1 and Mad2 were mainly localized in the pronuclei and started to become localized at the kinetochores at NEBD, clear localization was detected at the kinetochores at pro-metaphase, and the signal disappeared from the kinetochores at metaphase and anaphase/telophase (Fig. 1B and C). Moreover, co-localization of Bub3, BubR1, Mad2 and CREST was performed to further confirm the kinetochore localization of these proteins (Fig. 1D, E and F). These data imply that SAC may contribute to cell cycle surveillance as checkpoint proteins during the first cleavage of mouse embryos.

### Overexpression of SAC components inhibits metaphase-anaphase transition by preventing chromatid segregation in one-cell embryos

**Figure 2:**
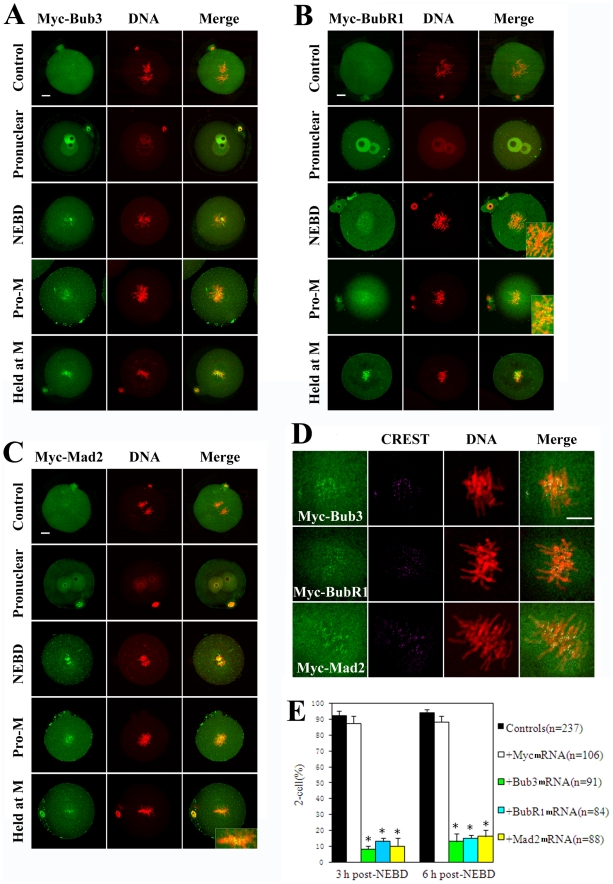
Overexpression of SAC inhibits metaphase-anaphase transition. (A) One-cell embryos at various stages were stained with anti-Myc-FITC antibody (green) to detect the overexpressed-Bub3 and with PI (red) to visualize DNA. Control and overexpression groups were injected with the same amount of Myc_6_ mRNA and Myc_6_-Bub3 mRNA. Clear signal of overexpressed Bub3 was observed at kinetochores in NEBD, pro-metaphase and metaphase stages. Overexpressed Bub3 arrested one-cell embryos at the metaphase stage by preventing sister chromatid segregation. (B and C) Overexpressed BubR1 or Mad2 also arrested one-cell embryos at the metaphase stage by preventing sister chromatid segregation. (D) Co-localization of overexpressed Bub3, BubR1, Mad2 and CREST at kinetochores. (E) Effect of overexpressed SAC on metaphase-anaphase transition. Percentage of 2-cell embryos is shown by mean±SEM. Different superscripts indicate statistical difference (p<0.05). Scale bars represent 10 µm.

To further characterize the role of SAC, Bub3, BubR1 and Mad2 overexpression experiments were performed. Pronuclear stage one-cell embryos were injected with Myc_6_-Bub3, Myc_6_-BubR1 or Myc_6_-Mad2 mRNA, followed by anti-myc-FITC antibody for immunofluorescent detection. The same amount of Myc_6_ mRNA was injected as control and resulted in no specific signal detection (Fig. 2, top of A, B and C). Myc_6_-Bub3 mRNA was successfully overexpressed within the embryos. In the pronuclear stage, diffused signal of overexpressed Bub3 was detected in the pronuclear area. In NEBD and pro-metaphase stages, overexpressed Bub3 was clearly localized at the kinetochores of chromosomes, which was similar to endogenous Bub3. During metaphase, however, overexpressed Bub3 was still present at the kinetochores and inhibited the transition from metaphase to anaphase by preventing sister chromatid segregation even though all chromosomes were aligned at the equatorial plane and were ready for the anaphase transition (Fig. 2A); the embryos could not overcome this inhibition even when cultured for 3 more hours (Fig. 2 E). In the BubR1 and Mad2 overexpression groups, most embryos were also unable to overcome this inhibition even if they were cultured for 3 more hours (approximately 6 hours post NEBD), indicating that overexpressed SAC inhibited the metaphase-anaphase transition (Fig. 2B, C and E). Co-localization of overexpressed Bub3, BubR1, Mad2 and CREST was performed to further confirm their localization at kinetochores (Fig. 2D).

### Deletion of SAC by RNAi accelerates the metaphase-anaphase transition and abrogates the metaphase arrest induced by nocodazole

**Figure 3:**
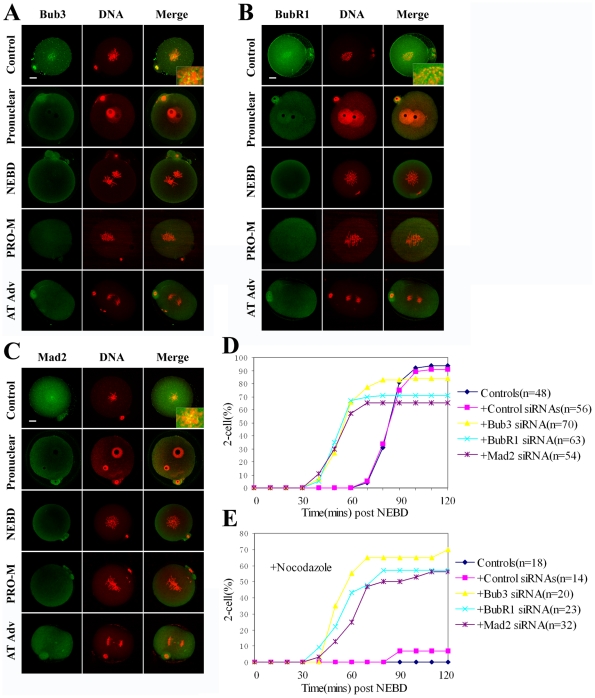
Deletion of SAC by RNAi accelerates the metaphase-anaphase transition and abrogates the metaphase arrest induced by nocodazole. (A) After RNAi, Bub3 signal disappeared from kinetochores, and anaphase was advanced. (B and C) Similar as in A, depletion of BubR1 and Mad2 also accelerates the metaphase-anaphase transition. (D) Normal embryos undergo metaphase-anaphase transition at approximately 90 min post-NEBD. However, in the RNAi group, most embryos completed this transition at approximately 60 min post-NEBD, indicating that depletion of SAC accelerated the metaphase-anaphase transition. (E) In the presence of nocodazole, mitosis in the control embryos was severely arrested. However, nocodazole treatment did not result in a significant alteration in SAC depleted embryos. ‘AT Adv’ refers to advanced anaphase or telophase. Scale bars represent 10 µm.

We further investigated the effects of SAC down-regulation on embryonic mitosis progression. Negative control siRNA or Bub3 siRNA was injected into metaphase II (MII) stage oocytes which were subsequently used for IVF. We first detected the RNAi efficiency by Western blot. As presented in Fig. S1 A, compared with the control group (control siRNA injection), Bub3 expression in siRNA-injected pronuclear stage embryos (14 hours after injection) was significantly reduced, revealing successful Bub3 down-regulation by RNAi. The expression level of Bub3 was unchanged for 3 hours of siRNA injection, thus it did not affect the process of second meiosis. The proteins of BubR1 and Mad2 were also successfully down-regulated by RNAi (Fig. S1 B and C). To further explore the role of Bub3, BubR1 and Mad2, we determined whether the metaphase-anaphase transition was effected by RNAi. As shown in Fig. 3A, in the control siRNA injection group, Bub3 signal was observed at kinetochores. However, in the Bub3 RNAi group, the Bub3 signal could not be detected at the pronuclear stage, and anaphase was advanced with no apparent metaphase stage. In normal cultures, most one-cell embryos underwent the metaphase-anaphase transition at approximately 90 min post-NEBD. However, in the RNAi group, most embryos completed this transition at approximately 60 min post-NEBD, indicating that depletion of the Bub3 accelerated the metaphase-anaphase transition. Depletion of BubR1 and Mad2 also accelerated the metaphase-anaphase transition as presented in Bub3 down-regulation group (Fig. 3B, C and D). To further assess whether the observed phenotype was caused by the breakdown of the spindle assembly checkpoint, one-cell embryos at the NEBD stage were incubated with 2 µM nocodazole, a microtubule depolymerizing drug, for approximately 3 hours. Under this treatment, 100% (18/18) of the control embryos and 93% (13/14) of the control siRNA injected embryos were arrested at one-cell stage (Fig. 3E). However, in the RNAi combined with nocodazole treatment experiment, 70% (14/20) of Bub3 depleted embryos, 57% (13/23) of BubR1 depleted embryos and 56% (18/32) of Mad2 depleted embryos were able to overcome this block and developed to 2-cell stage (Fig. 3E). These results indicate that SAC is essential for mitotic arrest of mouse embryos in response to spindle damage.

### Deletion of SAC by RNAi causes misaligned chromosomes and aneuploidy

**Figure 4:**
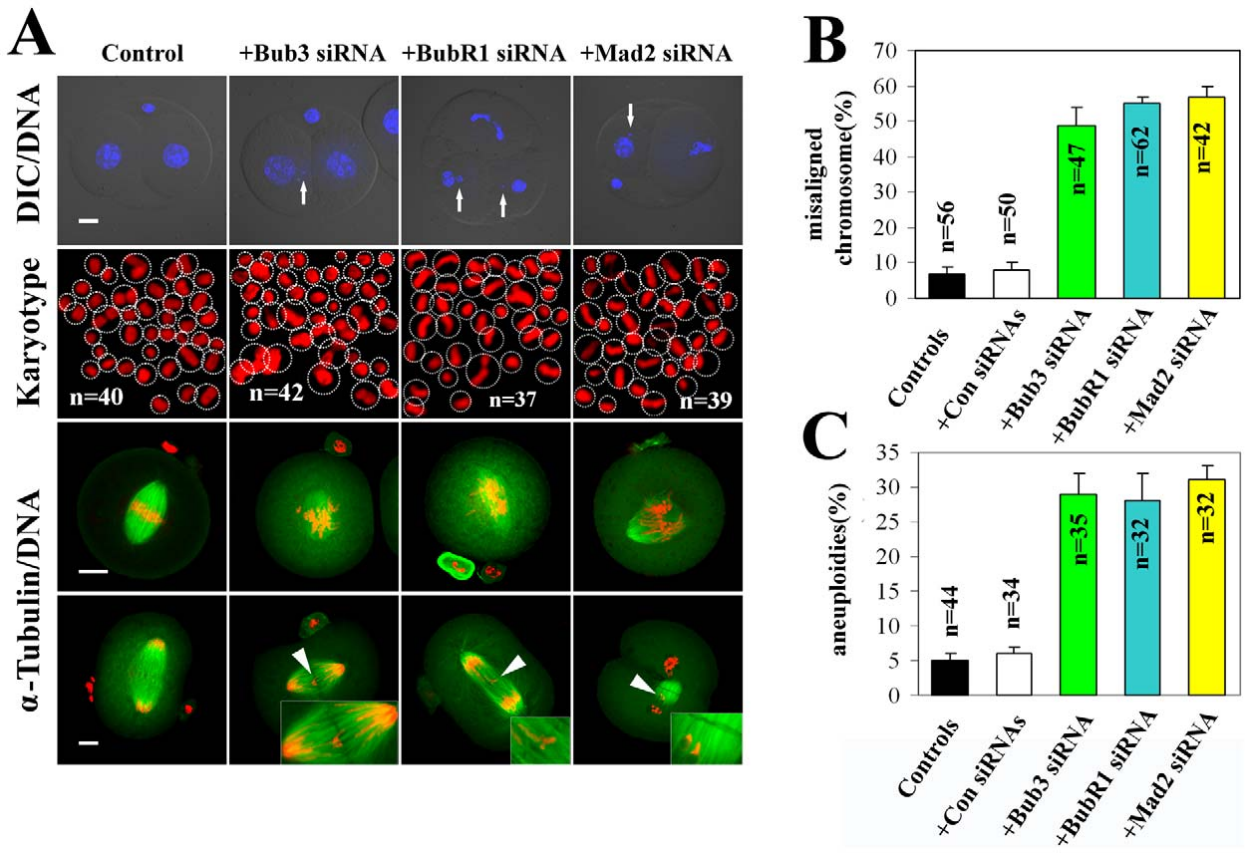
Deletion of SAC by RNAi causes misaligned chromosomes and aneuploidy. (A) Micronuclei (arrows) in embryos from SAC depleted embryos and chromosome spreading of SAC depleted embryos with chromosome numbers other than 40 (dotted circles) indicated aneuploidy. Chromosome misalignment was found in embryos from SAC depleted embryos, including both severe misalignment and lagging chromosomes, indicating the improper segregation in the absence of SAC. (B) Percentage of embryos with misaligned chromosomes. (C) Percentage of embryos with aneuploidies. Scale bars represent 20 µm.

The frequent observation of micronuclei in the SAC depleted embryos suggested a defect in chromosome stability (Fig. 4A). Thus, we examined the karyotype of the SAC depleted embryos. As anticipated, a significantly higher rate of aneuploidy was observed in Bub3, BubR1 or Mad2 down-regulated embryos compared to normal embryos (Fig. 4A and C). One critical event governing proper chromosome segregation is the spindle assembly checkpoint. To investigate whether the aneuploidy observed in the SAC depleted embryos was due to improper chromosome segregation, we imaged mitotic spindles and chromosomes in SAC down-regulated embryos. We observed a high incidence of chromosome misalignment in embryos from SAC depleted embryos by immunofluorescent microscopy, including both severe misalignment and lagging chromosomes (Fig. 4A and B), indicating improper segregation in the absence of SAC.

### Severe mitotic phenotypes and failure of nocodazole to arrest mitosis in SAC depleted blastocysts

**Figure 5:**
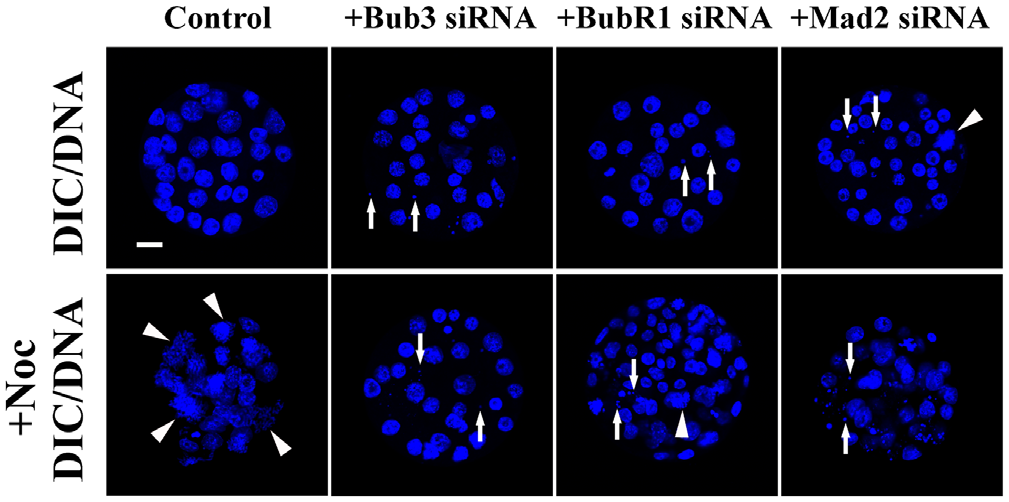
Severe mitotic phenotypes and failure of nocodazole to arrest mitosis in SAC depleted blastocysts. (Top) The SAC down-regulated embryos exhibited a significantly increased number of micronuclei (arrows) compared to the normal controls. (Bottom) In the presence of nocodazole, a significant increase in mitosis (arrowheads) was observed in normal controls. However, the treatment did not result in a significant alteration in mitotic indices of the SAC depleted embryos. Scale bars represent 20 µm.

To further examine the effects of SAC on subsequent divisions of preimplantation embryos, SAC down-regulated day 3.5 blastocysts were fixed and then stained with Hoechst 33342. We found that the SAC down-regulated embryos exhibited a significantly increased number of micronuclei compared to normal controls (Fig. 5). An average of 7–11 micronuclei per affected embryo was observed, compared with a baseline level of 0.6 micronuclei for normal controls (Table 1). The micronuclei seen in the affected embryos provided evidence for lagging chromosomes due to missegregation. No significant differences in the mitotic indices or cell number were seen between the affected embryos and normal controls (Table 1). To assess whether this observed phenotype was due to the down-regulation of SAC, day 3.5 embryos were incubated with 2 µM nocodazole for approximately 5 hours. The results indicated that mitosis in the normal embryos was severely arrested in the presence of nocodazole, as evident from a significant increase in mitosis from an untreated value of 6.5% to a treated value of 22.2% (Fig. 5 and Table 1). In contrast to this increase in mitotic indices, the treatment did not result in a significant alteration in the mitotic indices of the SAC down-regulated embryos compared to the untreated embryos (Fig. 5 and Table 1). Furthermore, following the treatment, a significant increase of the cell number in the SAC down-regulated embryos was also evidence for failure of nocodazole arrest (Table 1).

**Table 1:** Analysis of mitotic phenotypes at day 3.5 untreated or nocodazole-treated blastocysts from normal or SAC depleted embryos.

Untreated					+Nocodazole			
	Control	+Bub3 siRNA	+BubR1 siRNA	+Mad2 siRNA	Control	+Bub3 siRNA	+BubR1 siRNA	+Mad2 siRNA
No.of embryos	13	15	23	21	11	19	20	17
No.of cells^a^	34	33 (0.59)^b^	36 (0.27)	34 (0.29)	36	41(3.8×10^−2^)	45(1.4×10^−4^)	42(3.2×10^−3^)
Mitotic index^a^^,^ c	2.2^d^	1.8(0.21)	2.7(8.9×10^−2^)	1.5(1.3×10^−2^)	8^d^	2.1(1.3×10^−5^)	2.4(2.4×10^−5^)	1.8(1.1×10^−5^)
No.of micronuclei	0.6	7.2(1.1×10^−7^)	6.9(1.8×10^−13^)	10.7(4.9×10^−15^)	0.7	6.0(8.3×10^−12^)	8.8(8.4×10^−13^)	10.5(7.7×10^−12^)

a Average value per embryo.

b P values were derived using the student's t test.

c Expressed as percentage of cells in mitosis over total cell numbers.

d P = 4.1×10^−6^.

### Impaired developmental competence of SAC depleted embryos

Since the blastocysts in the above experiment exhibited increasing numbers of micronuclei or severe mitotic phenotypes, we undertook embryo transfer experiments to determine whether this will affect subsequent developmental competence. Five to ten blastocysts were transferred to each uterine horn of ICR mice. The embryos were allowed to develop *in vivo* to midgestation (9.5 dpc). The blastocysts transferred to pseudopregnant ICR females from SAC depleted embryos exhibited a significant decrease in implantation rate when compared to controls (87.2% for control embryos versus 54.2% for SAC depleted embryos, P<0.05; Table 2), as well as an increase in the percentage of implantation sites that were undergoing resorption (14.6% for control embryos versus 57.7% for SAC depleted embryos, P<0.01; Table 2). Although there was no difference in the percentage of embryos that were viable (91.4% for control embryos versus 90.9% for SAC depleted embryos, P>0.05; Table 2), the percentage of delayed development was quite high in SAC depleted group (14.3% for control embryos versus 90.9% for SAC depleted embryos, P<0.01; Table 2).

**Table 2:** Characteristics of control and SAC depleted litters following embryo transfer.

		No. of blastocysts transferred	No. of implantation sites (%)	No. of resorption sites (%)	No. of viable^a^ (%)	No. of delayed (%)
Control^b^	Litter 1	14	12(85.7)	2(16.7)	10(100)	1(10.0)
	Litter 2	16	13(81.3)	1(7.7)	11(91.7)	2(16.7)
	Litter 3	17	16(94.1)	3(18.8)	11(84.6)	2(15.4)
	Total	47	41(87.2)	6(14.6)	32(91.4)	5(14.3)
SAC depleted^c^	Litter 1	18	8(44.4)	4(50.0)	3(75.0)	4(100)
	Litter 2	17	11(64.7)	6(54.5)	5(100)	4(80.0)
	Litter 3	13	7(53.8)	5(71.4)	2(100)	2(100)
	Total	48	26(54.2) (1.4×10^−2^)^d^	15(57.7) (9.1×10^−3^)	10(90.9) (0.97)	10(90.9) (4.0×10^−3^)

a Viable embryos were defined as embryos having a heart beat, regardless of stage of development.

b Control refers to the embryos injected with control siRNAs.

c Embryos derived from litter 1, 2 and 3 were Bub3, BubR1 and Mad2 depleted, respectively.

d P values were derived using the student's t test.

## Discussion

Ensuring faithful chromosome separation during initial cell divisions is of critical importance in early embryonic development, where aneuploidy in even a few cells may have a devastating effect and lead to embryonic lethality. SAC is a surveillance system that senses failure of kinetochore attachment to microtubules and produces signals to hold the cell cycle at metaphase until all chromosomes attach to the spindle and congress to the metaphase plate [32]. Here we show that SAC is essential for the regulation of mitotic cell cycle progression in mouse cleavage stage embryos.

We first examined the expression and localization of these proteins, and found that SAC is localized at kinetochores during NEBD and pro-metaphase stages and maintains the localization until the metaphase-anaphase transition. The interesting behavior of SAC from ‘on’ to ‘off’ indicates that the retreat from kinetochores is a prerequisite for the embryos to enter anaphase, which exhibits the SAC protein's features as seen in mouse and human [29], [33]. This implies that SAC exerts a potential function after fertilization.

To characterize the role of SAC in early embryos, we conducted overexpression experiments. Overexpressed SAC evidently inhibited sister chromatid segregation, indicating that SAC is involved in monitoring the metaphase-anaphase transition. Previous studies found that in somatic cells and meiosis I mouse oocytes overexpression of SAC induced a metaphase arrest [29], [34]. The sensitivity of somatic cells to arrest at metaphase is graded, and approximately 10-fold overexpression of Mad2 above endogenous levels can induce this arrest, whereas at lower overexpression levels mitosis proceeds with normal kinetics [34]. In our overexpression study, the spindle checkpoint signals were still located to the kinetochores even if all chromosomes had aligned correctly at the equatorial metaphase plate, and most embryos were unable to overcome this inhibition even if they were cultured 6 hours post NEBD, 4 hours longer than needed for entrance into anaphase, indicating successful overexpression. We noted that a small number of one-cell embryos could not be inhibited at metaphase but entered anaphase. Indeed, not all embryos can express the exogenous mRNA smoothly or in time, thus the translated protein may not be sufficient or it may be sufficient but delayed in the recruitment to the kinetochores.

In HeLa cells, depletion of Mad2 shortens the duration of mitosis from 60 to 100 min and roughly halves the interval from NEBD to anaphase, leading to chromosome missegregation [12], [35]. To determine whether depletion of SAC would accelerate mitotic progression, we examined the duration of mitosis and found that it was approximately 30 min shorter in SAC-depleted embryos compared with controls. To further explore the role of SAC as spindle checkpoint proteins, we cultured one-cell embryos with nocodazole to determine whether the metaphase-anaphase transition was blocked by this drug. We found that in the presence of nocodazole, mitosis in the nocodazole-control embryos was severely arrested. However, nocodazole treatment did not result in significant alterations in SAC depleted embryos. Thus, depletion of SAC abrogates the metaphase arrest induced by nocodazole, which is consistent with findings in somatic cells and oocytes [14], [36], [37], further indicating that SAC functions as checkpoint in early embryos.

We surmised that the accelerated progression observed in embryos following depletion of SAC might result in aneuploidy. We therefore performed chromosome spreading and found that a large number of SAC depleted embryos exhibited incorrect numbers of chromosomes, which provided clear evidence for aneuploidy. In fact, the actual rates of aneuploidy may even be underestimated, because only one blastomere of the two-cell embryos was used for chromosome spreading to avoid mixture of chromosomes from two blastomeres. The vast majority of control embryos displayed normal chromosome alignment with only approximately 1/14 being misaligned. In contrast, following SAC depletion, misalignment was found in approximately half of the embryos. Of these embryos, some were severely misaligned, whereas others displayed a few lagging chromosomes. Mitosis in somatic cells may provide an explanation for this phenotype. Following 30% Mad2 depletion, HeLa cells fail to arrest at metaphase in the presence of nocodazole and prematurely inactivate Cdk1 [23], [35]. Following 90% reduction in Mad2 levels, however, spindle assembly and chromosome condensation are seriously perturbed, cyclin B is degraded and mitosis is shortened by approximately 40% [35]. PtK_1_ cells injected with Mad2 antibodies enter into anaphase prematurely but assemble normal spindles, indicating that a substantial level of Mad2 function remains [10], [35]. Thus, the different phenotypes observed in SAC depleted embryos are a graded response to differing degrees of loss of SAC function. SAC proteins have been reported to exert their function interdependently, thus depletion of a single SAC protein is sufficient to affect the metaphase-anaphase transition. Consistent with our findings, the phenotype of Bub3 and Mad2 genetic-disrupted embryos share many features, both mutants show chromosome segregation errors and fail to arrest in the presence of nocodazole [13], [14].

In order to further understand the role of SAC in cleavage stage embryos, we created SAC depleted blastocysts. Determination of cell number indicated that mitotic division in the SAC depleted blastocysts did not change at day 3.5. However, the embryos accumulated such abundance of mitotic errors, as evidenced by the formation of micronuclei. In previous studies on the Mad and Bub genes, microtubule-depolymerizing drugs have been used to assess whether the spindle assembly checkpoint is compromised in the mutants. These mutants fail to arrest in response to spindle damage, resulting in chromosome missegregation. We have used a similar strategy to determine whether SAC functions as mitotic spindle checkpoint in the cleavage stage mouse embryos. We found that in the presence of nocodazole, normal embryos are severely arrested in mitosis, as indicated by a large increase in mitotic indices, compared to untreated normal embryos. However, the mitotic index in SAC depleted embryos remains unaffected when they are challenged with nocodazole. This observation indicates that the SAC depleted embryos can escape the block imposed by the mitotic spindle checkpoint pathway.

In summary, we show here that the SAC is essential for normal mitosis of cleavage stage mouse embryos. Specifically, deletion of SAC components causes micronuclei formation, chromosome misalignment and aneuploidy. Our results may help to understand how missegregation events during cleavage can ultimately lead to the generation of aneuploidy or spontaneous abortions in humans.

## Materials and Methods

All chemicals and media were purchased from Sigma Chemical Company (St. Louis, MO) except for those specifically mentioned.

### Ethics Statement

Mice care and handling were conducted in accordance with the Animal Research Committee guidelines of the Institute of Zoology, Chinese Academy of Sciences. The institute does not issue a number to any animal study, but there is an ethical committee to guide animal use. Each study requires the permit to use animals from the committee, and this study was approved by the Animal Research Committee of the Institute of Zoology, Chinese Academy of Sciences. The animal facility must get licensing from the experimental animal committee of Beijing city. The animal handling staff (including each post-doc and doctor student) must be trained before using animals. Mice were housed in a temperature-controlled room with proper darkness-light cycles, fed with a regular diet, and maintained under the care of the Laboratory Animal Unit, Institute of Zoology, Chinese Academy of Sciences. The mice were killed by cervical dislocation. The only procedure performed on the dead animals is the collection of oocyte from the oviduct.

### Oocyte and embryo collection and culture

Six to eight-week-old ICR females were superovulated by injection of 5 IU of pregnant mares' serum gonadotropin (PMSG), followed 48 h later by 5 IU of human chorionic gonadotropin (hCG). The oocytes were recovered from the oviducts 17–18 hours post-hCG, and the cumulus cells were dispersed with 1 mg/ml hyaluronidase. Sperm were expelled from the cauda epididymis of male mice into 200 µl human tubal fluid (HTF) medium and incubated under mineral oil for 1–2 hours at 37°C for capacitation. A sperm suspension at a concentration of 6–7×10^5^ sperm/ml was used to inseminate eggs in a 200 µl drop of HTF medium under mineral oil. After coincubation with sperm for 6 hours, the inseminated eggs were washed and cultured in 50-µl droplets of potassium simplex optimized medium (KSOM) under mineral oil at 37°C in humidified air containing 5% CO_2_. The formation of pronuclei was examined at 12 hours after insemination, and only those oocytes that extruded second polar bodies and formed pronuclei were used for further analyses. Starting from 15 to 16 hours of insemination embryos were checked every 10 minutes to determine the time for NEBD and then collected for further analyses at different times of NEBD. Embryos undergoing NEBD were defined as 0 min. Embryos were cultured in KOSM to NEBD (0 min), pro-metaphase (60 min), metaphase (90 min), anaphase (110 min) and 2-cell stages (140 min). Blastocysts were collected at 3.5 days after fertilization. For embryo transfer experiments, SAC depleted blastocysts were collected and immediately transferred to 2.5 dpc pseudopregnant ICR females. Females were sacrificed at 9.5 dpc relative to the recipient female and concepti were immediately collected.

### Plasmid constructs

Total RNA was extracted from 150 mouse zygotes using RNeasy micro purification kit (Qiagen), and the first strand cDNA was generated with cDNA synthesis kit (Takara), using poly (dT) primers. The primer sequences were described previously [38], [39] except for the full length of Mad2 (GenBank accession no. U83902, forward, 5′ -TCAGGCCGGCCGATGGCACAGCAGCTCGCCCGAG- 3′; reverse, 5′ - GTTGGCGCGCCTCAGTCATTGACAGGAATTTTG- 3′). To detect the overexpressed protein, the CDS of these genes were then NH_2_-terminally Myc_6_-tagged. For *in vitro* transcription reactions, the Myc_6_-Bub3 was subcloned into the modified pRN3p vector (a gift from Dr. Jie Na, Harvard University), and the Myc_6_-BubR1 and Myc_6_-Mad2 were subcloned into the pCS2 plus vector as described previously [32], [33].

### RNA synthesis

The Myc_6_-Bub3-pRN3p plasmid was linearized by *Sfi* I, and the Myc_6_-BubR1-pCS2+ and the Myc_6_-Mad2-pCS2+ plasmid were linearized by *Sal* I. The linearized plasmid was purified by gel extraction kit (Qiagen). The mMessage mMachine (Ambion) was used to produce capped mRNA which was purified using the RNeasy cleanup kit (Qiagen). The concentration was detected by Beckman DU 530 Analyzer, and diluted into 2.5 mg/ml for final microinjection.

### Microinjection of mRNA or siRNA

Microinjections were performed using a Nikon Diaphot ECLIPSE TE 300 (Nikon UK Ltd., Kingston upon Thames, Surrey, UK) inverted microscope equipped with Narishige MM0-202N hydraulic three-dimensional micromanipulators (Narishige Inc., Sea Cliff, NY) and completed within 30 minutes. Approximately 2.5 mg/ml Myc_6_-Bub3 mRNA, Myc_6_-BubR1 mRNA or Myc_6_-Mad2 mRNA was injected into the cytoplasm of pronuclear stage zygotes and expression of protein was completed within 2–2.5 hours. The same amount of Myc_6_ mRNA was injected as control.

Small interfering RNAs (siRNA) of Bub3, BubR1 and Mad2 (Ambion) were microinjected into the cytoplasm of MII oocytes before IVF to deplete protein of these genes. The sequences of the siRNAs were: Bub3, UGCACGAUUUGAACACUGATT; BubR1, AGCCAAGGAAUUGGCGUUUTT; Mad2, CAGCAUUUUGUAUCAGCGUTT. 25 mM siRNAs were used for each gene, and the same amount of negative control siRNA was injected as control.

### Immunoblotting analysis

Mouse embryos at the appropriate stages and embryos injected with siRNAs were collected in sodium dodecyl sulfate (SDS) sample buffer and boiled for 5 minutes. Immunoblotting was performed as described previously [40]. Briefly, the proteins were separated by sodium dodecyl sulfate polyacrylamide gel electrophoresis (SDS-PAGE) and then electrically transferred to polyvinylidene fluoride membranes. Following transfer, the membranes were blocked in Tris-buffered saline Tween-20 (TBST, TBS containing 0.1% Tween 20) containing 5% skimmed milk for 2 hours, followed by incubation overnight at 4°C with anti-Bub3 (BD Biosciences), anti-BubR1 (Abcam), anti-Mad2 (Santa Cruz) or anti-myc (Invitrogen) with dilutions of 1∶500, 1∶1000, 1∶1000, and 1∶500, respectively. After washing in TBST, the membranes were incubated for 1 hour at 37°C with 1∶1000 horseradish peroxidase (HRP) -conjugated IgG. To detect β-actin, the membranes were washed in the washing buffer (100 mM β-mercaptoethanol, 20% SDS, and 62.5 mM Tris, pH 6.7) for 30 minutes at 55°C. β-actin was then assayed on the same membrane by using anti-β-actin antibody (1∶1000) and HRP-conjugated IgG. Finally, the membranes were detected by the enhanced chemiluminescence detection system (Amersham, Piscataway, NJ).

### Immunofluorescent microscopy, chromosome spreading and nuclear staining

Immunofluorescence was performed as described previously [40]. For single staining of Bub3, Myc_6_-Bub3, BubR1, Myc_6_-BubR1, Mad2, Myc_6_-Mad2 and α-tubulin, embryos were fixed in 4% paraformaldehyde in phosphate buffered saline (PBS, pH 7.4) for at least 30 minutes at room temperature. After being permeabilized with 0.5% Triton X-100 at room temperature for 20 minutes, embryos were blocked in 1% bovine serum albumin (BSA)-supplemented PBS for 1 hour and incubated overnight at 4°C with 1∶50 anti-Bub3 (BD Bioscience), 1∶25 anti-BubR1 (Abcam), 1∶50 anti-Mad2 (Santa Cruz), 1∶200 anti-Myc-FITC (Invitrogen), or 1∶200 anti-α-tubulin-FITC antibodies, respectively. After three washes in PBS containing 0.1% Tween 20 and 0.01% Triton X-100 for 5 minutes each, the embryos were labeled with 1∶100 FITC-conjugated IgG for 1 hour at room temperature. After washing in PBS containing 0.1% Tween 20 and 0.01% Triton X-100, the embryos were co-stained with propidium iodide (PI; 10 µg/ml in PBS) or Hoechst 33342 (10 µg/ml in PBS). Finally, the embryos were mounted on glass slides and examined with a confocal laser scanning microscope (Zeiss LSM 510 META, Germany). For chromosome spreading, one blastomere of the two-cell embryos was used to avoid mixture of chromosomes from two blastomeres. Embryos were left for 15 minutes in 1% sodium citrate at room temperature and then fixed with fresh methanol: glacial acetic acid (3∶1). 10 µg/ml PI was used for chromosome staining. For nuclear staining, embryos were fixed in 4% paraformaldehyde for 30 min, followed by Hoechst 33342 (10 µg/ml in PBS) for 10 min. Cells were examined with a confocal laser scanning microscope (Zeiss LSM 510 META, Germany). Instrument settings were kept constant for each replicate.

## Supporting Information

Figure S1 **Down-regulation of SAC by RNAi.** (A) Samples from control and RNAi groups were collected to test the efficiency of Bub3-RNAi. Control refers to the embryos injected with control siRNAs. The protein was down-regulated 14 hours after siRNA injection, but unchanged for 3 hours post siRNA injection, indicating that it did not affect the process of second meiosis. (B and C) Similar as in A, the efficiency of BubR1-RNAi and Mad2-RNAi was tested. Both proteins exhibited low expression levels at 14 hour of siRNA injection, whereas no changes were observed at 3 hour of siRNA injection.
(TIF)
