# Supplementary figures and images for: Spindle Assembly Checkpoint Regulates Mitotic Cell Cycle Progression during Preimplantation Embryo Development
Source: PLoS One. 2011 Jun 24;6(6):e21557. doi: 10.1371/journal.pone.0021557 (PMC3123354; doi:10.1371/journal.pone.0021557)

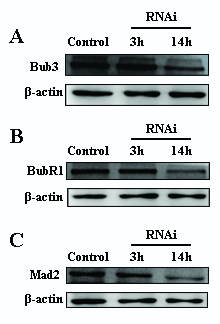

Supplement: Figure S1 — Down-regulation of SAC by RNAi. (A) Samples from control and RNAi groups were collected to test the efficiency of Bub3-RNAi. Control refers to the embryos injected with control siRNAs. The protein was down-regulated 14 hours after siRNA injection, but unchanged for 3 hours post siRNA injection, indicating that it did not affect the process of second meiosis. (B and C) Similar as in A, the efficiency of BubR1-RNAi and Mad2-RNAi was tested. Both proteins exhibited low expression levels at 14 hour of siRNA injection, whereas no changes were observed at 3 hour of siRNA injection. (TIF) [file pone.0021557.s001.tif]
